# Understanding Behavior in Phelan-McDermid Syndrome

**DOI:** 10.3389/fpsyt.2022.836807

**Published:** 2022-05-26

**Authors:** Annemiek M. Landlust, Linda Visser, Boudien C. T. Flapper, Selma A. J. Ruiter, Renée J. Zwanenburg, Conny M. A. van Ravenswaaij-Arts, Ingrid D. C. van Balkom

**Affiliations:** ^1^Autism Team Northern-Netherlands, Jonx, Department of (Youth) Mental Health and Autism, Lentis Psychiatric Institute, Groningen, Netherlands; ^2^Department of Genetics, University Medical Centre Groningen, Groningen, Netherlands; ^3^Leibniz Institute for Research and Information in Education (DIPF), Frankfurt am Main, Germany; ^4^Center for Research on Individual Development and Adaptive Education of Children at Risk, Frankfurt am Main, Germany; ^5^Department of Paediatrics, University Medical Centre Groningen, Groningen, Netherlands; ^6^De Kinderacademie Groningen, Groningen, Netherlands; ^7^Department of Psychiatry, Rob Giel Research Centre, University Medical Center Groningen, Groningen, Netherlands

**Keywords:** Phelan-McDermid syndrome, neurodevelopmental phenotype, 22q13 deletion syndrome, behavioral difficulties, intellectual disability, contextual assessments

## Abstract

**Background:**

Phelan-McDermid syndrome (PMS) or 22q13.3 deletion syndrome is a rare genetic disorder characterized by developmental delay, hypotonia and severely delayed speech. Behavioral difficulties are often reported in PMS, although knowledge of behavioral profiles and the interpretation of reported behavior remains limited. Understanding the meaning of behavior requires considering the context as well as other domains of functioning, for example the individual's level of cognitive, social and emotional development. Combining structured direct in-person neurodevelopmental assessments with contextual assessments to enable meaningful interpretations of reported behavior on functional dimensions across multiple units of analysis, as proposed by the RDoc framework, is essential.

**Methods:**

In this article we present a structured multidisciplinary method of assessment through direct in-person neurodevelopmental assessments and assessment of contextual factors. Our study sample includes data of 33 children with an average age of 6.2 years (range 1.1 to 15.7) with PMS, obtained through individual in-person assessments in combination with parent informed questionnaires. We assessed developmental age using the Bayley-III, adaptive behavior was assessed with the Vineland screener, social-emotional development with the ESSEON-R and behavior by using the CBCL.

**Results:**

Our results show a great deal of variability in phenotypic presentation with regard to behavior, symptom expression and symptom severity in individuals with PMS. The data on behavior is interpreted in the context of the individual's level of cognitive, adaptive development and the (genetic) context. Behavioral data showed high levels of withdrawn behavior and attention problems. More than half of the children showed borderline or clinical symptoms related to Autism Spectrum Disorder (ASD).

**Conclusions:**

The interpretation of the meaning of certain behavior in PMS is often based on questionnaires and descriptions without taking the specific context of development into account. Combining questionnaires with direct in-person assessments measuring different domains of functioning should be considered a more accurate method to interpret the meaning of findings in order to understand behavior in rare genetic disorders associated with developmental delay such as PMS. Direct in-person assessment provides valuable and specific information relevant to understanding individual behavior and inform treatment as well as increase knowledge of the neurodevelopmental phenotype in individuals with PMS. More specific application of the proposed frameworks on behavior in PMS is desirable in making useful interpretations.

## Background

Phelan McDermid syndrome (PMS) or 22q13 deletion syndrome is a rare genetic disorder characterized by developmental delay, hypotonia and absent or severely delayed speech (1, 2). Specific behavioral issues (3, 4), minor physical anomalies, seizures (1, 3) and sleep disorders (5, 6) are often described in children and adults with PMS. Previous studies have shown that intellectual disability is a prominent feature of PMS and is mostly severe to profound (4, 7–9). Studies of neurodevelopmental and behavioral aspects in PMS however have often used assessments, methods, and tools more suited to assess mild to moderate intellectual disability (ID) (10). Behavior is often interpreted dichotomously as present or absent, but is rarely considered within the dimension of ID or psychosocial context. Soorya et al. (10) suggested a framework for assessing individuals with rare genetic disorders and Profound Intellectual and Multiple Disabilities (PIMD) and suggested PMS as an example of PIMD due to the severe to profound intellectual disability. Assessing neurodevelopmental aspects and behavior in PIMD, such as PMS, would therefore require a multidisciplinary and multimodal neuropsychological assessment. The framework suggested by Soorya et al. (10) is in line with the framework of Research Domain criteria (RDoc) proposed by the National Institute of Mental Health (NIMH) in 2009 (11). The RDoc framework aims at a better understanding of mental health issues opposed to current models like DSM-5 by the American Psychiatric Association. The RDoc criteria provide a framework that focuses on the full dimensional aspects of behavior and understanding behavior within the context rather than a description of psychopathological behavior being present or absent in an individual or group. The RDoc framework is an integrative model of different constructs within five domains that interact and are necessary to understand the meaning of behavior through multiple mechanisms. Domains within the RDoc are Negative valence systems, Positive valence systems, Cognitive systems, Systems for social processes and Arousal/modulatory processes. The RDoc framework focusses on underlying psychological constructs instead of systemizing behavior on a symptomatic level.

For example the degree of ID and other developmental domains as suggested in the RDoc criteria have a profound effect on behavior (12) and therefore on the interpretation of developmental and behavior measures in PIMD such as PMS. Esteves et al. (12) shows the correlation between adaptive functioning and behavioral problems including behavioral aspects of autism in individuals with ID. In PMS the delay in behavioral or social-emotional development is often more severe than would be expected based on the individuals' cognitive capabilities (4).

Oliver et al. (13) emphasize the importance of studying distinctive behavior in relation to the developmental perspective of specific groups of individuals with ID in their studies of phenotypes in specific syndromes. In PMS distinctive behavior has been described generally, but rarely within the perspective of the developmental delay. The developmental perspective on behavior in specific groups contributes not only to our understanding of behavior within that group but also on the possible etiology of this behavior in non-syndromic groups. For example expectations on mood regulation would differ enormously between a 3 year old child and a thirteen year old child. In the thirteen year old, tantrums can be a symptom of an oppositional defiant disorder, from the developmental perspective of a 3 year old, tantrums are normal behavior.

In 34 children with PMS between 0.7 and 14.8 years of age, Zwanenburg et al. (8) found that the average developmental level increased up to the calendar age of approximately 6 years, but not thereafter.

In this paper we reconsider data in part (Bayley-III and Vineland) previously described by Zwanenburg et al. (8) from the perspectives of the renewed frameworks suggested by Soorya et al. as well as the RDoc domains of functioning. We combined this previously described data with data on behavior and functioning gathered in the same timeframe. We advocate the use of the described perspectives on interpretation of behavior in rare genetic disorders such as PMS and propose adaptations in assessment of behavior that will enhance possibilities for interpretation. Domains of functioning described in this article are cognitive development, adaptive behavior, social-emotional development and behavior.

Zwanenburg et al. (8) found that the maximum developmental age equivalent (DAE) of the 34 children in this study was approximately 3 years, with one exception of a developmental level of 4.5 years.

Adaptive behavior can be described as everyday life skills on domains such as social, communication, motor and practical daily skills. Previous studies on adaptive behavior in children with PMS (14–16) had sample sizes ranging from 18 to 40 and age ranges across the three studies between 2 and 18 years (with one exception of 42 years) (15). The results showed adaptive behavior in the below-average range on all domains, with relatively high scores in the motor domain and low scores in the communication domain (14–16). Comparable results were found in another study of seven adults with PMS (7).

Behavioral problems associated with PMS are also described in persons with severe to profound ID and/or Autism Spectrum Disorder (ASD) but without PMS, e.g., mouthing behavior, social problems and stereotypies (3). Shaw et al. (15) found increased levels of mainly internalizing and maladaptive behavior, while other studies have found aggressive behavior and self-injury occurring in a little over 40% of people with PMS (6, 16). Self-injury, like hitting or biting oneself, seems to be associated with impulsivity and often serves the purpose of self-stimulation (16). Rahman (17) performed a study in 46 individuals with PMS between 2 and 27 years of age, both with and without ASD, and found few problems in the areas of anxiety, self-esteem and somatoform behavior in the whole sample. In a relatively large study involving 201 individuals with PMS between 0 and 64 years of age, behavioral difficulties appeared to decrease with age (18). In adults, difficulties in the areas of social relationships and anxiety are more prominent (7).

Vogels et al. (19) reviewed literature on behavior in PMS and found multiple psychiatric issues such as catatonia, bipolar disorder and ASD associated with PMS. ASD rates seemed to be depending on type of assessment. The rate of ASD characteristics is estimated up to 94% (20, 21). In a study involving 71 individuals with PMS between 0 and 40 years of age (*M* = 7.5, *SD* = 2.5), Sarasua et al. (20) found that 26% of participants older than 3 years of age had ASD. In a study of 201 individuals (18), ASD characteristics appeared to increase with age, from 19% in 3- to 4.9-year-olds to 60% in those over 18 (average 31%). Other psychiatric issues diagnosed in PMS are ADHD, psychosis and depression, and bipolar disorder (3, 15, 22). In adults, psychosis seems to occur more frequently than ASD (15). The average age of onset of psychiatric symptoms is between 15 and 20 years, but the range is large (4, 22). The study by Rahman (17) among 46 individuals found that comorbidity of PMS and ASD was related to greater impairment in adaptive behavior in the areas of socialization and communication.

Social-emotional development is described as learning how to relate to the social world and be able to differentiate, express and perceive emotions. Specific patterns of social-emotional development and behavior have been reported in specific genetic disorders like Down syndrome or Williams syndrome (23). In PMS little is known about the social-emotional development, only specific behavior like social communication are described. Size of deletions has been suggested to be related to level of development and behavior in children with PMS, but there is a large inter-individual variability (20). More severe developmental delay in the language, motor and cognitive domains appears to be associated with larger deletion sizes. This was found in the study by Zwanenburg et al. (8), the studies by Sarasua et al. (18, 20) and in a third study by Sarasua et al. (24) involving 79 individuals between 0 and 40 years of age (*M* = 7.7).

In this study we describe the findings on behavior, developmental domains and deletion size in the same sample of 33 children with PMS previously described by Zwanenburg et al. (8). We suggest modifications and a structured multidisciplinary approach in assessing and interpreting behavior and development in rare genetic disorders and PIMD such as PMS. Such a structured modified approach based on RDoc criteria and the framework Soorya et al. (10) proposed, leads to understanding the meaning of behavior in children with PMS. This informs interventions on care for individuals with PMS and allows comparison in behavior and levels of functioning within and between syndromes.

## Methods

### Participants and Procedure

The sample included 33 children with PMS, due to a deletion 22q13.3, who were diagnosed at the University Medical Centre Groningen or had been referred from other medical centers in the Netherlands. This study examines data from the same sample described in Zwanenburg et al. (8), with the exclusion of child number 7 in that study. This child had a mosaic deletion, which is not comparable to the other deletion types. Zwanenburg et al. (8) previously described a subset of the data, the Bayley-III and VABS in their descriptive article. In this study we analyzed the data on behavior and social-emotional development and compared these results with the previously described data on the Bayley and VABS. The data on behavior and social-emotional development have not been published previously. This data was collected within the same timeframe as the previously published data on the Bayley-III and the VABS.

Our study population (see Table 1 for details) consisted of 8 boys and 25 girls with an average age of 6.2 years (range 1.1 to 15.7). Twenty-eight children had a simple terminal deletion, with three also having an additional copy number variation. The other five children had a 22q13.3 deletion due to a ring chromosome 22. The average deletion size was 3.9 Mb (range 0.2–9.2 Mb). The calendar age presented is the age at the date of the Vineland test administration, which leads to slight differences with the age mentioned in our previous paper, which was based on the day of the Bayley-III assessment (see Section *Instruments*). An educational psychologist assessed the child in a familiar setting. A more detailed description of the sample and procedure can be found in Zwanenburg et al. (8). Characteristics of our study sample are shown in Table 1.

**Table 1:** Characteristics of the children in the sample.

**ID no**.	**Age (mo)**	**Sex**	**Deletion type**	**Deletion size (Mb)**	**Walking unassisted (mo)**	**Medication at 1st assessment**
1	13	M	Terminal	6.5	25 (crawling)	None
2	16	F	Terminal	2.1	12	None
3	22	F	Terminal	1.9	19	None
4	26	F	Terminal	7.7	36	Salbutamol (as needed)
5	22	F	Terminal + dup 13q (2.3 Mb)	7.3	39 (walking assisted)	None
6	17	F	Terminal	9.2	12 (rolling over)	None
8	37	F	Terminal	3.2	30	Valproic acid (for absence like periods)
9	37	M	Terminal	2.1	17	None
10	39	F	Terminal	182 kb	16	Not reported
11	41	F	Terminal	587 kb	24	Macrogol and omeprazole
12	42	F	Terminal	6.2	27	None
13	45	F	Terminal	6.6	20	None
14	45	F	Terminal	7.4	76	None
15	46	F	Terminal	6.2	25	None
16	47	F	Terminal + del 16p (761 kb)	3.0	42	Beclometason dipropionate, salbutamole, ipratropium bromide
17	47	M	Terminal	182 kb	18	Risperidone and clonidine
18	64	F	Terminal	183 kb	16	Macrogol
19	65	F	Ring 22	2.3	23	None
20	82	F	Terminal	1.6	17	None
21	96	M	Ring 22	3.1	28	None
22	99	F	Ring 22	3.4	31	None
23	92	M	Ring 22	2.7	24	None
24	92	F	Terminal^c^	n.a.	n.a.	Not reported
25	110	M	Terminal	6.1	43	Melatonin
26	105	F	Terminal	6.4	96	Omeprazole, alginic acid, domperidone, trimethoprim, melatonin
27	119	F	Terminal	377 kb	16	Melatonin
28	112	F	Terminal + dup 12q (5.1 Mb)	2.0	22	None
29	123	M	Terminal	7.8	48	None
30	129	F	Ring 22	3.4	32	None
31	118	F	Terminal	224 kb	15	None
32	142	M	Terminal	5.0	24 (walking assisted)	Alimemazine and melatonin
33	157	F	Terminal	3.5	32	None
34	188	F	Terminal	5.7	19	Lamotrigine (for fever-induced convulsions)

*no, number; mo, months*.

### Instruments

We assessed cognitive development, using the Dutch Bayley Scales of Infant and Toddler Development, third edition (Bayley-III) (25). This instrument contains subscales for cognition, receptive and expressive language, and fine and gross motor development, which are assessed using a standardized in-person test administration. The test contains norms for children up to 42 months of age and is also used for older children with a developmental level up to 42 months. The test results of the children in the current sample were previously reported in Zwanenburg et al. (8).

The Dutch Vineland Screener 0-6 (26) an adaptation of the Vineland Adaptive Behavior Scales (VABS) (27) assesses adaptive behavior based on caregiver-report. This instrument can also be used for older children with a developmental level up to 6 years. The Vineland contains subscales for communication, social behavior, daily skills and motor skills. The parent indicates to what extent the child displays each of 72 descriptions of behavior using a 3-point Likert scale (yes, usually / sometimes or partially / no, never) and an additional response option “unknown.” The raw score can be converted to a developmental age equivalent (DAE) ranging from 6 to 70 (communication), 1–70 (social behavior), 10–68 (daily skills), 0–58 (motor skills), and 2–68 (adaptive behavior total score) months.

We used the Dutch Child Behavior Checklist for children of 1.5–5 years (CBCL) (28), a questionnaire for assessing internalizing (anxiety/depression, somatic, withdrawn) and externalizing (attention problems, aggression) behavior. In addition, the CBCL yields scores for five problem areas, namely affective problems, anxiety, ASD (named *pervasive developmental disorder* in the CBCL), ADHD and oppositional deviant disorder (ODD). It contains 100 items with short descriptions of behavior for which the respondent indicates if this suits the child on a 3-point Likert scale (not at all / a bit or sometimes / clearly or often). The questionnaire yields *t*-values (*M* = 50, *SD* = 10) for each subscale, for internalizing and externalizing problems, and for each of the five problem areas. *T*-values between 65 and 70 are in the borderline range. *T*-values above 70 are in the clinical range, indicating behavioral problems.

For all analyses involving the CBCL, we included all children with a calendar age of 18 months or older because the target group of the instrument starts at this age (28). As the CBCL can also be used for children with ID (29), we did include children with a developmental age below 18 months, after verifying that the descriptive results did not differ considerably from those of the children with a higher developmental level.

The ESSEON-R (30) is a questionnaire with 76 short descriptions of behavior for assessing social-emotional development of children with a developmental level between 0 and 14 years. The questionnaire yields a DAE per domain as well as a total DAE. The ESSEON-R is explicitly meant for assessing the social-emotional development of children with intellectual impairments or psychiatric problems.

All questionnaires were proxy questionnaires filled in by one or both parents or a care worker who was very familiar with the child and subsequently evaluated with parents or care workers by an educational psychologist. Deletion sizes were evaluated by a clinical geneticist. General principles on assessment as proposed in the framework of Soorya et al. (6) were applied in the assessments.

### Data Analysis

Because of the small sample size, all the analyses reported in the current paper are descriptive in nature. We used both SPSS (Version 23) (31) and R (32) for the statistical analyses.

First an overview was made of the scores of the children in the sample on the tests and questionnaires. Second, we described the domains of the Vineland, ESSEON-R and CBCL on which the children obtained the highest scores. We visualized and/or described differences between subgroups of children based on scores on the Bayley-III, developmental level or deletion size. Regarding deletion size, we used the same groupings used in Zwanenburg et al. (8): <225 kb, 225 kb−6.7 Mb and >6.7 Mb. The reason for this size grouping is that children with a very small deletion have a higher developmental level, on average. The higher boundary of 6.7 Mb is downstream of the *PARVB* gene. Children were divided in two groups of calendar age with a cut-off at 6 years (72 months) based on the results of the Bayley-III ceiling effect described in Zwanenburg (8). The DAE on the Bayley-III cognition scale could be equally distributed in 2 comparable groups (*n* = 12 and *n* = 13) with a DAE of ≤ vs. >18 months. We also visualized the relationship between general behavior on the CBCL and adaptive behavior.

For visualizing behavioral characteristics as well as differences between subgroups therein, we used line graphs both for the individual children and for the group mean, so that individual differences would be reflected in the results [R Package “ggplot2” (33)]. Developmental age equivalents (DAE) in the figures are based on the cognitive domain of the Bayley-III.

As sex differences have not been found in the developmental or behavioral characteristics of persons with PMS (6), we did not take them into account.

## Results

Characteristics of our study sample are shown in Table 1. Table 2 shows the scores of the children on cognition (Bayley-III), adaptive behavior (Vineland) and social-emotional development (ESSEON-R). Table 3 shows the scores regarding behavior (CBCL). For a small number of children, data on the Bayley-III (*n* = 1), Vineland (*n* = 3), or CBCL (*n* = 4) were missing.

**Table 2:** Developmental Age Equivalents (DAE) in months of cognition (Bayley-III), adaptive behavior (Vineland), and social-emotional development (ESSEON-R).

	**Bayley-III**	**Vineland**					**ESSEON-R**		
**ID**	**Cognition**	**Communication**	**Social skills**	**Daily skills**	**Motor skills**	**Sum score**	**Social**	**Emotional**	**Total**
1	3	6	6	10	2	4	6	6	6
2	8	9	12	18	20	13	30	18	24
3	8						12	12	12
4	5	8	6	14	14	9	18	12	15
5	13	11	10	10	6	7	30	18	24
6	1						12	12	12
8	7	6	19	10	17	12	18	18	18
9	13	16	24	12	29	19	30	12	21
10	23	18	17	25	30	22	48	36	42
11	8	9	3	12	17	9	6	24	15
12	16	14	15	10	14	12	12	18	15
13	22	16	28	25	24	22	30	24	27
14	7	11	17	14	7	11	12	24	18
15	18	14	23	28	27	22	36	24	30
16	24	14	14	18	20	15	36	24	30
17	22	18	21	23	30	22	36	24	30
18	38	33	28	39	30	32	72	36	54
19	25	28	39	34	37	34	48	36	42
20	22	18	12	41	37	26	12	24	18
21	21	21	26	32	38	29	36	18	27
22	21	19	24	21	29	23	30	24	27
23	25	19	12	23	30	20	36	18	27
24	8	6	3	12	20	9	12	18	15
25	10	13	15	16	15	13	30	24	27
26	6	6	8	12	7	7	3	3	3
27	24	6	10	19	25	14	6	12	9
28	21	8	14	14	22	13	12	6	9
29	14	6	8	16	17	10	6	18	12
30	30	38	23	27	24	27	36	24	30
31	52						72	84	78
32	7	9	12	14	4	8	18	24	21
33	25	23	37	34	27	30	30	24	27
34		9	6	12	12	8	6	12	9
Mean	17.1	14.4	16.4	19.8	21.0	16.7	25.4	21.5	23.5
sd	10.9	8.2	9.3	9.1	10.0	8.5	17.7	13.8	14.8

**Table 3:** Scores on behavioral problems measured with Child Behavior Checklist (CBCL).

**CBCL (** ***t-*** **values)**												
**Subscales**						**Main scales**		**Problem areas**				
**ID**	**Anxiety/ Depression**	**Somatic complaints**	**Withdrawn**	**Attention problems**	**Aggressive**	**Internalizing**	**Externalizing**	**Affective**	**Anxiety**	**PDD**	**ADHD**	**ODD**
3	51	50	79	67	50	63	51	63	57	72	50	50
4	50	62	67	51	50	56	44	52	50	63	50	51
5	64	62	76	67	65	71	66	79	59	74	57	70
8	51	53	67	77	62	56	66	60	54	63	64	59
9	52	53	76	62	56	65	58	52	63	77	54	52
10	51	67	63	70	53	64	58	60	57	75	60	52
11	50	50	94	77	51	63	57	75	50	77	60	51
12	50	53	70	70	51	56	56	63	50	63	60	50
13	71	79	85	57	72	78	69	79	75	84	60	64
14	50	58	63	53	59	60	58	67	54	70	57	50
15	50	50	63	67	56	51	59	51	50	60	51	55
16	50	50	67	51	50	47	39	56	50	57	50	50
17	67	76	70	73	84	78	82	75	73	81	72	80
18	51	58	56	62	62	58	62	52	57	60	57	55
19	51	53	63	57	55	58	56	67	54	63	52	55
20	52	58	70	80	72	70	77	63	54	79	67	70
22	50	50	67	67	64	53	65	51	51	67	57	55
26	50	70	73	67	50	62	47	56	50	72	57	50
27	50	53	73	67	51	62	54	72	50	77	57	50
28	50	50	82	53	50	56	42	60	50	72	51	50
29	52	50	67	80	69	66	74	70	63	77	64	64
30	50	62	63	57	52	61	54	60	59	74	57	50
31	59	76	76	73	65	71	68	75	70	72	67	67
32	52	53	56	57	50	62	47	50	59	63	54	50
33	51	58	63	50	50	56	42	52	50	63	50	50
34	50	50	70	57	50	56	48	52	50	68	51	51
M	52.6	57.0	68.3	63.4	56.9	59.2	56.3	60.9	55.5	68.3	56.7	55.3
sd	5.4	8.8	9.7	9.3	8.9	10.3	11.7	9.6	7.2	8.8	5.9	7.9

*PDD, pervasive developmental disorder; ADHD, attention deficit hyperactivity disorder; ODD, oppositional deviant disorder; M, Mean; sd, standard deviation; Clinical threshold = >70*.

### Adaptive Behavior

**Figure 1:**
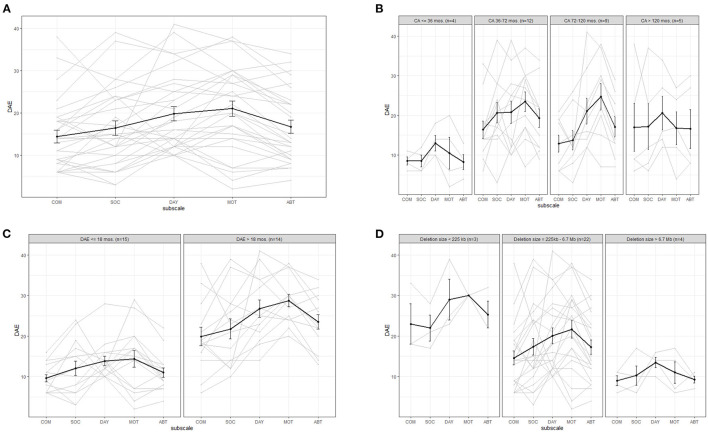
Average (bold line) and individual behavior profiles on the Vineland for **(A)** the whole sample and for subgroups based on **(B)** calendar age (CA), **(C)** developmental age equivalent based on cognition scale in the Bayley-III (DAE) and **(D)** deletion size. COM, communication; SOC, social behavior; DAY, daily skills; MOT, motor skills; ABT, adaptive behavior total score. Please take into account: the average calendar age was 74 months (6.2 years).

Figure 1 shows the individual as well as the average scores on the Vineland subscales. The average developmental age equivalent (DAE) for adaptive behavior was 17 months, with a range of 4 to 34 months. This wide range is partly explained by differences in calendar age. However, based on the DAEs shown in Table 2 for the children with identification number 20 and higher, who all have a calendar age > 6 years, the inter-individual variation still is large. Variation is also large with respect to the direction and magnitude of the differences between the subscale scores. The level ranges from about 14 to 21 months between the subscales, with the highest averages for motor skills and daily skills. However, the subscale daily living skills has a relatively high baseline level, as the lowest possible DAE is 10, which influences the average. On the individual level, the subscale levels range from 2 to 42 months. A total of 13 of the children had their highest subscale score on motor skills, seven on daily skills, six on social skills and two on communication.

Comparing the Vineland profiles of children younger than 6 years old to children older than 6 years, did not show any clear differences. We therefore included a graph comparing four different age groups based on developmental milestones, see Figure 1B. Although the groups are small, this graph shows that the average developmental level in the area of adaptive behavior does increase with increasing age from 3 years up, the increase in adaptive behavior seems to plateau at the age of 6 years. Figure 1C illustrates the adaptive behavior profiles for children with a cognitive developmental level (as measured with the Bayley-III cognition scale) up to vs. >18 months. If the cognitive DAE is higher, the DAE for adaptive behavior is also, on average, higher. Figure 1D compares children with different deletion sizes, and a clear trend of decreasing level of adaptive behavior with increasing deletion size can be seen. We also compared children with different deletion types and found that children with a ring 22 deletion (median deletion size: 2.98 Mb) have slightly higher average levels of adaptive behavior (subscale averages ranging between 25 and 32 months) than children with a terminal deletion.

### Behavior

**Figure 2:**
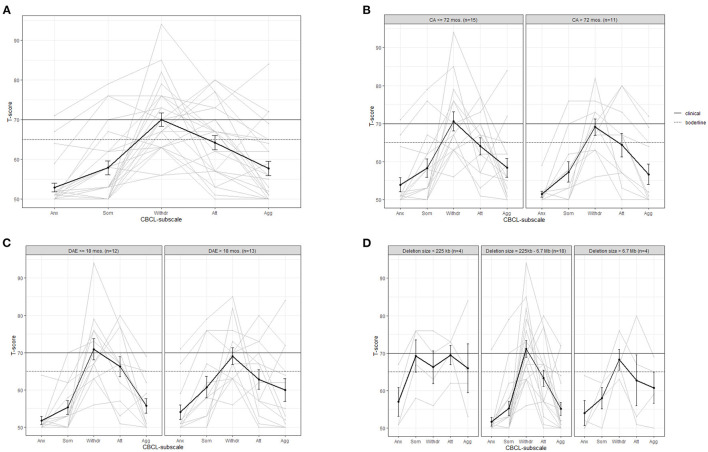
Average (bold line) and individual behavior profiles on the CBCL subscales for **(A)** the whole sample and for subgroups based on **(B)** calendar age (CA), **(C)** developmental age equivalent (DAE) in months based on cognition scale Bayley-III and **(D)** deletion size. Anx, anxiety/depression; Som, somatic; Withdr, withdrawn; Att, attention problems; Agg, aggression.

Figure 2 shows the behavioral profiles of the 26 children >1.5 years of age on the basis of the CBCL subscale *t*-values for behavior. Compared to the Vineland results, the inter-individual variation is large. As can be seen in Figure 2A, withdrawn behavior shows both the highest average *t*-value (in the borderline range) and the highest individual *t*-value (*t* = 94). For all other subscales, the average *t*-values are below the borderline range. On an individual level, two children scored in the borderline or clinical range for anxiety, five for somatic symptoms, 18 for withdrawn behavior, 14 for attention problems and six for aggression. Most children have the highest *t*-value for withdrawn behavior (*n* = 15) or attention problems (*n* = 6).

Figure 2B shows that there is no large difference between children up to vs. above 6 years old in terms of behavior. Figure 2C shows that in children with a DAE above 18 months on the Bayley-III cognitive scale, scores on somatic problems and aggression are slightly higher than under 18 months DAE. The group with a DAE under 18 months show a *t*-value within clinical range for attention problems opposed to the group above 18 months DAE. In both groups the *t*-value for withdrawn behavior is the highest score and within clinical range. Figure 2D compares children with different deletion sizes. Children with a deletion size below 225 kb have clearly higher average *t*-values for somatic problems, attention problems and aggression, which are all in the borderline range. When looking at deletion type, children with a ring 22 deletion (*n* = 3) have slightly lower average levels of behavioral problems (subscale averages below the borderline level, ranging between 50 and 64) than children with a terminal deletion (subscale averages: 53 to 75).

**Figure 3:**
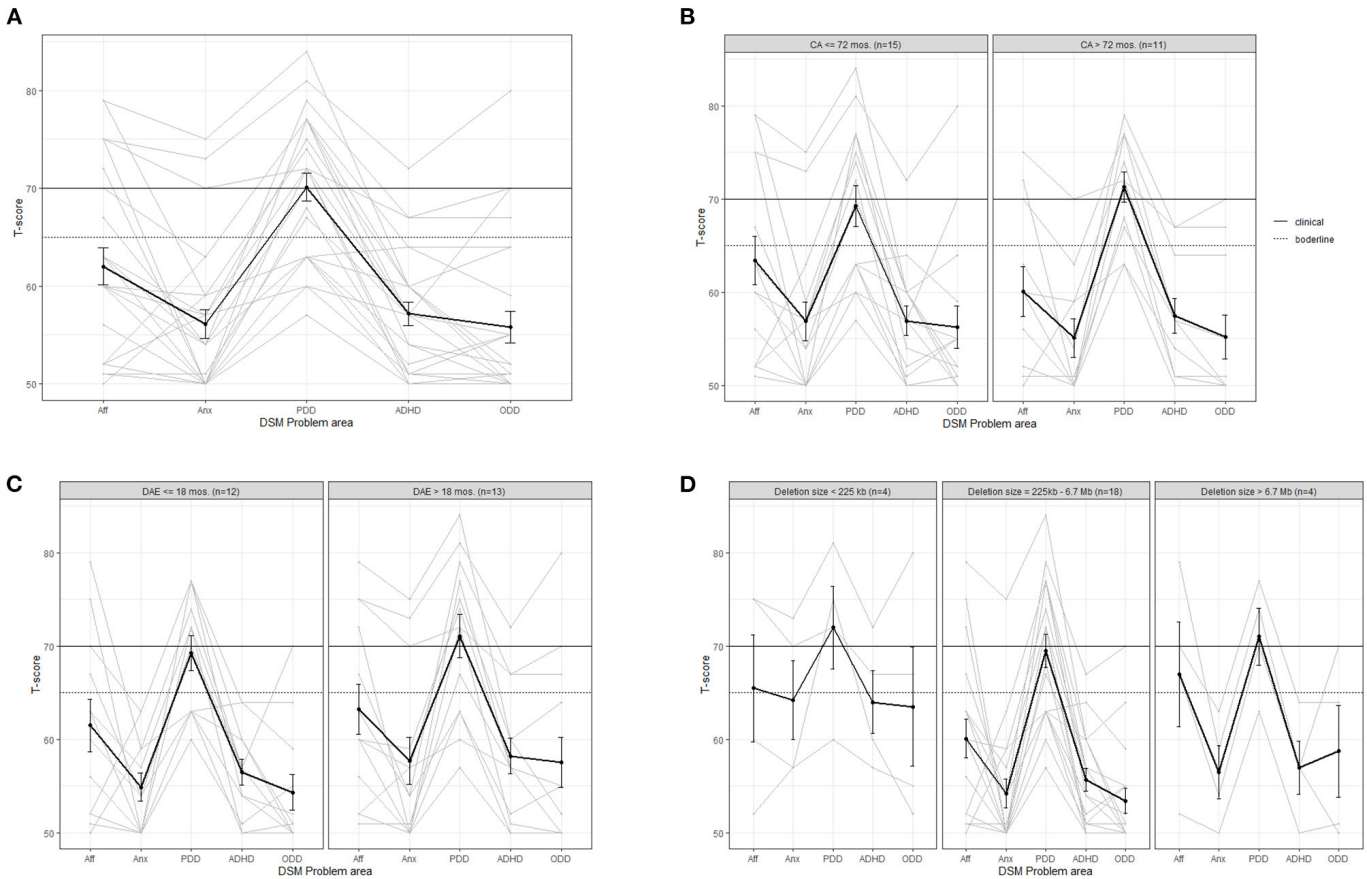
Average (bold line) and individual behavior profiles on the CBCL DSM problem areas for **(A)** the whole sample and for subgroups based on **(B)** calendar age (CA), **(C)** developmental age equivalent (DAE) in months based on cognition scale Bayley-III and **(D)** deletion size. Aff, affective problems; Anx, Anxiety; PDD, pervasive developmental disorder; ADHD, attention deficit hyperactivity disorder; ODD, oppositional deviant disorder.

Figure 3 shows the behavioral profiles for the CBCL-scales based on classification areas of the Diagnostic and Statistical Manual of Mental Disorders (DSM). The *t*-value for pervasive developmental disorder (PDD) has the highest average (*t* = 70) and the highest individual value (*t* = 84). All other average *t*-values are below the borderline range. On an individual level, five children scored in the borderline or clinical range for affective problems, three for anxiety, 17 for PDD, three for ADHD and four for ODD. Most children have the highest *t*-value for PDD (*n* = 21).

No clear differences can be observed between younger and older children (Figure 3B) or between children with a lower or higher cognitive developmental level (Figure 3C), although the variation in PDD-scores is clearly lower in children who are above 72 months of age. The average *t*-value for anxiety, ADHD and ODD is just below the borderline range for children with a deletion size below 225 kb, which is higher than that of children with larger deletion sizes. However, this subgroup is very small (*n* = 4, see Figure 3D). We found no clear differences when comparing children with different deletion types.

### Social-Emotional Development

**Figure 4:**
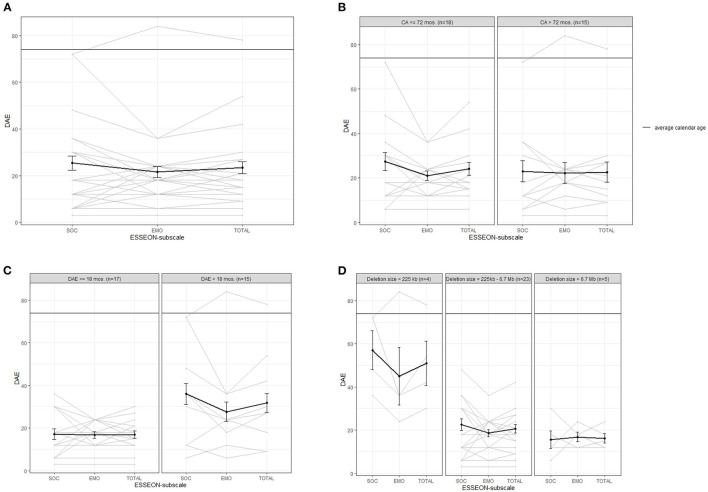
Average (bold line) and individual behavior profiles on the ESSEON-R for **(A)** the whole sample and for subgroups based on **(B)** calendar age (CA), **(C)** developmental age equivalent (DAE) in months based on the Bayley-III cognition scale and **(D)** deletion size. SOC, social development; EMO, emotional development.

Figure 4 shows the levels of social and emotional development of the children, which is around 22 to 25 months, on average. The graphs of the individual children show large variation. The levels of social behavior do differ slightly from the levels of emotional behavior within the children. With the exception of one child with a clearly higher level of social behavior.

Figure 4B shows no clear differences between younger and older children. Among the children with a cognitive developmental level above 18 months there are more children with higher levels of social and emotional behavior (above 30 months) than in the group with a lower cognitive DAE (see graph c). Children with a deletion size smaller than 225 kb have higher levels of social and emotional development than children with larger deletion sizes, although the group is small (*n* = 4), see Figure 4D. We found no clear differences between children with a ring 22 vs. a terminal deletion.

### ASD Symptoms and Adaptive Behavior

**Figure 5:**
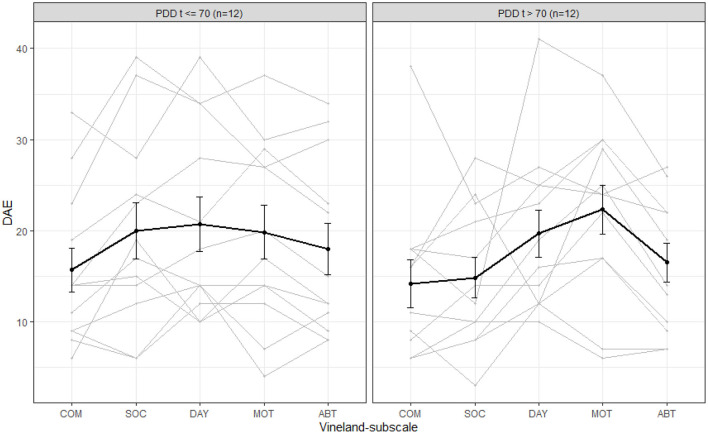
Average (bold line) and individual behavior profiles on the Vineland for children with and without CBCL-scores in the clinical range for pervasive developmental disorder (PDD). COM, communication; SOC, social behavior; DAY, daily skills; MOT, motor skills; ABT, adaptive behavior total score; DAE, developmental age equivalent in months based on Bayley-III cognition scale. Please take into account: the average calendar age was 74 months (6.2 years).

Children with a dual diagnosis of ID with ASD in general show a lower level of adaptive behavior on the social domain compared to intellectually disabled children without ASD (34). Figure 5 shows the level of adaptive behavior, comparing children with and without a CBCL *t*-value in the clinical range (*t* > 70) on the PDD-subscale (indicating possible ASD). In children without a *t*-value in the clinical range, their profile is relatively balanced, with an average DAE around 20 months, except for the communication subscale, for which the average is around 16 months. Children with a PDD *t*-value within the clinical range also have a lower score in the social domain, on average. The average level of communication skills does not differ much between the groups, but if we were to exclude child 30 (communication DAE = 38 months, PDD *t*-value = 74), this average would be lower in the group of children with PDD *t*-values in the clinical range. The average level of adaptive behavior in the domains daily living skills and motor skills does not differ much between the two groups. Within the group with clinical PDD *t*-values, fewer scores above the level of 30 months are obtained than in the other group.

## Discussion

This study followed the structured modified approach based on RDoc criteria and the proposed framework by Soorya et al. (10). We found large variations in cognitive development, adaptive behavior and social-emotional development. To understand the meaning of our results we interpreted developmental levels and behavior within the other domains of functioning. Cognitively the children showed severe developmental delay given the average calendar age of 6.2 years (range 1.11 to 15.7). The highest levels of adaptive behavior were found in the areas of motor skills and daily skills. The level was 17 months, on average, with a range of 4 to 34 months. The wide range of adaptive behavior can only partially be explained by differences in calendar age: levels of adaptive behavior seem to increase until the calendar age of approximately 36 months, and then appear to even out. Levels of adaptive behavior appeared higher with higher levels of cognitive development and smaller deletion sizes, which is consistent with a previous finding that relatively small deletions were related to a more favorable developmental phenotype (15).

The large variability and the observation of higher levels of adaptive behavior in children with smaller deletion sizes are also consistent with the earlier study into the development of children with PMS (8). We could not confirm previous observations of increased adaptive behavior than could be expected based on their cognitive developmental level (4, 5), except for the communicative domain. This last result could, however, be affected by limitations related to the validity of the communication domain (see paragraph adaptive behavior, communication below).

Regarding behavior, we found that withdrawn behavior, followed by attention problems were most frequent. The parents reported relatively little anxiety, which is in line with previous research (17). Although previous research in a sample including both children and adults described behavioral difficulties decreasing with age (18), we found no clear trend with respect to calendar age, which could be due to the limited age range up to 15 years and/or our small sample size. A higher cognitive developmental level and a smaller deletion size seem to be related to higher levels of somatic symptoms and aggression (*n* = 4). The increased average scores on these specific subscales could be specific to PMS.

Considering behavioral issues ASD-symptoms were most frequent, whereas small deletion size was related to increased symptoms of anxiety, ADHD and ODD. The level of reported ASD-symptoms is in line with previous findings (20, 21). Our findings of increased problems in the areas of withdrawn behavior, attention and ASD are comparable to those of a study in children with ASD (35). Of course, the fact that ASD-like symptoms co-occur with ID associated with PMS requires careful consideration whether these symptoms are more intense and frequent than expected for level of ID and merit an ASD diagnosis. Interestingly, our results show that few of the children with PMS show ASD symptom scores in the borderline or clinical range. Therefore, an additional ASD diagnostic trajectory can be helpful in understanding the meaning of behavior and differentiating between children and to identify underlying care needs.

High levels of supposed ASD symptoms were related to lower levels of adaptive behavior in the social domain. This is according to expectations of adaptive behavior in children with confirmed ASD diagnosis. All the children in the sample had somewhat lower levels in the communication domain, independent of their level of ASD symptoms and understandable within the context of the ID. As children with PMS have impaired language abilities (4, 5), this could explain the fact that the scores on the Vineland items measuring communication skills. These depend, to a large extent, on the verbal language skills of the children (e.g., “Does he/she have a vocabulary of at least 50 recognizable words?” or “Does he/she speak in full sentences?”), whereas this is not the case for the items measuring social skills (e.g., “Does he/she play with a toy or object, alone or with others?”). Possibly, the Vineland underestimates non-verbal communicative adaptive behavior in children with PMS in that the lower scores reflect low levels of expressive verbal language and do not sufficiently take into account non-verbal communication abilities.

The level of social-emotional behavior was around 23 months on average, but also showed a large inter-individual variation. A higher cognitive developmental level was related to higher levels of social-emotional behavior, but this was only true for the subgroup of children with a small deletion size.

Consistent with earlier results (14, 18), smaller deletions and higher cognitive developmental level were related to higher levels of adaptive behavior, but also to more specific problems (somatic problems, attention problems, aggression and anxiety). This is unexpected, given the finding that lower intellectual ability level is related to having more CBCL scores in the deviant range (36). It may be that children who have a higher level of adaptive behavior are more aware of their limitations, are exposed to higher expectations due to their performance in adaptive behavior and therefore experience more stress, which could be expressed in the form of maladaptive behavior. Behavior like aggression and anxiety is very difficult to recognize in children with a younger developmental stage or age. Aggressive behavior is to some extent normal in younger stages of development and anxiety is related to developmental stage as well. Cognitive capacities are needed to comprehend possible danger and experience anxiety. Children with a higher developmental level might be better able to express themselves, this is also the case in expressing emotions. However, this hypothetical relationship between adaptive behavior and behavior is not clearly reflected in our results on the basis of the total score for internalizing and externalizing behavior.

The recognition and interpretation of behavior in children with lower developmental levels is also relevant in a more general sense in children with PMS and could play an explanatory role in our findings as proposed by the framework of Soorya et al. (10). The relatively high levels of withdrawn behavior and ASD symptoms in the sample were partly based on results on items describing behavior that can also be explained by the low level of cognitive and language development of children with PMS. More specifically, the items for which the answer category *clearly or often* was chosen most often (for more than half of the children) were: “Acts too young for his/her age,” “Does not respond when others talk to him/her” and “Speech problems.” This suggests that our results may include an overestimation of withdrawn behavior and ASD symptoms in these children. Children with a higher cognitive developmental level showed higher levels of somatic symptoms and aggression in our sample, which could imply that somatic symptoms and aggression are not easily recognizable in children with lower developmental levels (4, 7, 17) (Table 4). Even in a study using general principles for assessments in PIMD, as proposed in the framework of Soorya et al. (10) and the functional domains as proposed by the RDoc framework, interpreting behavior in PIMD such as PMS remains challenging.

**Table 4:** Previously published frequent somatic problems in PMS.

**Frequent somatic problems in PMS**
Hypotonia
Low pain perception
Sleep problems
Constipation
Regulating body temperature
Swallowing
Vision problems
Epilepsy

### Limitations and Directions for Future Research

The main limitation is the small sample size which is directly related to the rarity of PMS. The small sample and even smaller subgroup sizes mean that the results are highly sensitive to sampling variation and no firm conclusions can be drawn. However, our study adds to the body of evidence on development and behavior in children with PMS. A second important limitation is that the validity of the Vineland, CBCL and ESSEON-R for children with a PIMD as PMS has not yet been explicitly studied, and there are reasons to suspect measurement non-invariance. This means that the test validity might not be optimal for children with PMS in comparison to children without PMS and the same behavior (e.g., the measured construct). Previous research results about the validity of the CBCL for children with intellectual disability are inconsistent: one study found measurement invariance (29), while another found measurement invariance on the level of the total test score, but not for the subscales (37). As studying measurement invariance in relation to PMS is difficult due to the rarity of the syndrome, the descriptive results underpin the careful study and description of behavioral phenotype in PMS. In this study the general principles of assessment in PIMD (10) are applied in the assessments of the functional domains, but we argue these should also be applied in assessment and interpretation of behavior. The multi units of analysis based on the RDoc framework were used in this study and these should also be used when interpreting behavior in a PIMD as PMS. For example, expressive language (cognitive systems in RDoc framework) could be of influence on the scores regarding behavior. Our study endorses more explicit application of the other RDoc domains like negative valence, positive valence and arousal systems when assessing and interpreting behavior in PIMD like PMS. These domains could be of great use when interpreting documented behavior in children and adults with PMS.

When it comes to the CBCL, Koskentausta et al. (38) indeed found that the CBCL is less reliable to assess psychopathology in children with moderate, severe or profound intellectual disability, although this conclusion was based on descriptive statistics only. Another limitation is the fact that we used the CBCL, which can be used in children 1.5 years of age and up, although our sample included younger children. We solved this by excluding the children below 1.5 years of age from the analyses involving the CBCL, which means we cannot draw conclusions about the behavioral problems of these youngest children with PMS. In this study profiles of the scores have been analyzed instead of individual scores. The use of profiles limited the use of the individual data but provide interpretation on possible underlying developmental aspects.

Future research should not only be focused on replicating the results in situations in which larger samples of children with PMS can be formed, studies should ideally use instruments that are widely used internationally so that data from multiple studies in various countries can be combined to overcome the problems related to small sample sizes. Research based on longitudinal data would also be valuable, all the more so because this would help overcome the sample size challenge by collecting more information per child. This would also help to answer questions about how the behavior develops over time within children. In addition, having multiple assessments per child enhances the reliability of the data in total because an unreliable assessment due to, for example, tiredness during testing can be identified if the results deviate greatly from the results of other assessments in the same child. The framework of Soorya et al. and the RDoc framework should be taken into account when assessing functional domains or behavior in children with PIMD.

### Implications for Daily Practice

Our results have important implications for understanding behavior in PMS and adjusting the surroundings for children with PMS. Lower developmental levels and language skills are often reflected in difficulties adapting and responding to the environment, leading to stress. Stress reduces the possibilities for development, increases behavioral problems and decreases quality of life. Early identification of difficulties makes it possible to stimulate development and offer suitable support, to allow children with PMS to benefit from their environment. Difficulties need to be identified at an early stage so that suitable support can be given in the years where the children show the largest possibilities for development and emergence of more severe problems can be prevented. Children with PMS may need extra support in developing their expressive communication skills, for instance using visual communication or specific communication treatment programs.

Greater awareness of difficult to understand behavioral issues, psychiatric problems and underlying unmet needs, particularly in children with a low developmental level, is needed so measures can be taken to improve developmental opportunities and recognize unmet needs. In children with a small deletion size, the risk for behavioral problems in areas other than ASD is also increased.

### Conclusions

Our results show a large variation between children with PMS in terms of adaptive behavior, behavior and social-emotional development. Moreover, large intra-individual differences were found between the various domains. Contrary to the general understanding, average levels of adaptive behavior in our sample were not lower than, but rather consistent with levels of cognitive development. Levels of adaptive behavior were highest in the areas of motor and daily skills. Levels of adaptive behavior seem to increase up to the calendar age of approximately 36 months, and then seem to level out. Problems were mainly found in the areas of withdrawn behavior, followed by attention problems. In children with a small deletion size, symptoms of anxiety, ADHD and ODD seem to be increased. Interpreting psychiatric symptoms and behavior in an PIMD such as PMS remained challenging despite the use of available frameworks. Specific diagnostic assessment with the use of valid instruments for the level of ID is very important. Findings should be interpreted by an multidisciplinary team.

A small deletion size seems to be related to higher levels of adaptive behavior and social-emotional development. The frequency of ASD symptoms appeared not to be related to deletion size. High levels of ASD symptoms seem related to lower levels of adaptive behavior in the social domain.

Altogether, these results add to those of earlier studies and help to define the development and behavior of children with PMS. The small subgroup sizes, large inter-individual variability, and the potentially limited validity of the assessments need to be taken into account when interpreting the results. The findings underline the importance of neuropsychological and behavioral assessments within the frameworks of PIMD and RDoc domains when it comes to interpreting behavior in PMS. Early identification and interventions in expressive communication within the context of developmental level could be helpful to optimize early developmental opportunities, prevent stress and prevent the emergence of specific behavioral problems in children with PMS.

## Data Availability Statement

The raw data supporting the conclusions of this article will be made available upon request by the authors, without undue reservation.

## Ethics Statement

The studies involving human participants were reviewed and approved by Medical Ethical Review Board of the University Medical Center Groningen. Written informed consent to participate in this study was provided by the participants' legal guardian/next of kin.

## Author Contributions

RZ collected the clinical data, conducted the data preparation, and started with the data analysis. LV analyzed the data and drafted the manuscript. SR supervised developmental and behavior assessments, collected the data, and commented on the manuscript. BF collected pediatric clinical data, contributed to data interpretation, and commented on the manuscript. CvR-A coordinated the project and commented on the manuscript. AL and IvB interpreted all results, drafted, and finalized the manuscript. All authors read and approved the final manuscript.

## Funding

The collection of the data that formed the basis for the current study was supported by grants from the Netherlands Organization for Health Research and Development (ZonMw 113-20-2009 to RZ and CvR-A and ZonMw 15701.3002 to SR). ZonMw had no involvement in the writing of this paper or the decision to submit the paper for publication.

## Conflict of Interest

The authors declare that the research was conducted in the absence of any commercial or financial relationships that could be construed as a potential conflict of interest.

## Publisher's Note

All claims expressed in this article are solely those of the authors and do not necessarily represent those of their affiliated organizations, or those of the publisher, the editors and the reviewers. Any product that may be evaluated in this article, or claim that may be made by its manufacturer, is not guaranteed or endorsed by the publisher.

## Abbreviations

PMS: Phelan-McDermid syndrome
ASD: autism spectrum disorder
ADHD: attention deficit hyperactivity disorder
PDD: pervasive developmental disorder
ODD: oppositional deviant disorder
DAE: developmental age equivalent
CBCL: Child Behavior Checklist
Bayley-III, Bayley Scales of Infant and Toddler Development, third edition: Dutch Version
DSM: Diagnostic and Statistical Manual of Mental Disorders.
